# Oesophagectomy rates and post-resection outcomes in patients with cancer of the oesophagus and gastro-oesophageal junction: a population-based study using linked health administrative linked data

**DOI:** 10.1186/1472-6963-12-384

**Published:** 2012-11-08

**Authors:** Efty P Stavrou, Robyn Ward, Sallie-Anne Pearson

**Affiliations:** 1 Lowy Cancer Research Centre, Prince of Wales Clinical School, University of New South Wales, Sydney, NSW, Australia; 2 Faculty of Pharmacy, University of Sydney, Sydney, NSW, Australia; 3 Kolling Institute of Medical Research, University of Sydney, St Leonards, NSW, 2065, Australia

**Keywords:** Hospital outcomes, Oesophageal cancer, Administrative health data, Data linkage

## Abstract

**Background:**

Hospital performance is being benchmarked increasingly against surgical indicators such as 30-day mortality, length-of-stay, survival and post-surgery complication rates. The aim of this paper was to examine oesophagectomy rates and post-surgical outcomes in cancers of the oesophagus and gastro-oesophageal junction and to determine how the addition of gastro-oesophageal cancer to oesophageal cancer impacts on these outcomes.

**Methods:**

Our study population consisted of patients with a primary invasive oesophageal or gastro-oesophageal cancer identified from the NSW Cancer Registry from July 2000-Dec 2007. Their records were linked to the hospital separation data for determination of resection rates and post-resection outcomes. We used multivariate logistic regression analyses to examine factors associated with oesophagectomy and post-resection outcomes. Cox-proportional hazard regression analysis was used to examine one-year cancer survival following oesophagectomy.

**Results:**

We observed some changes in resection rates and surgical outcomes with the addition of gastro-oesophageal cancer patients to the oesophageal cancer cohort. 14.6% of oesophageal cancer patients and 26.4% of gastro-oesophageal cancer patients had an oesophagectomy; an overall oesophagectomy rate of 18.2% in the combined cohort. In the combined cohort, oesophagectomy was associated with younger age, being male and Australian-born, having non-metastatic disease or adenocarcinoma and being admitted in a co-located hospital. Rates of length-of-stay >28 days (20.9% vs 19.7%), 30-day mortality (3.8% vs 2.7%) and one-year survival post-surgery (24.5% vs 23.1%) were similar between oesophageal cancer alone and the combined cohort; whilst 30-day complication rates were 21.5% versus 17.0% respectively. Some factors statistically associated with post-resection complication in oesophageal cancer alone were not significant in the overall cohort. Poorer post-resection outcomes were associated with some patient (older age, birthplace) and hospital-related characteristics (fiscal sector, area health service).

**Conclusion:**

Outcomes following oesophagectomy in oesophageal and gastro-oesophageal cancer patients in NSW are within world benchmarks. Our study demonstrates that the inclusion of gastro-oesophageal cancer did alter some outcomes compared to analysis based solely on oesophageal cancer. As such, care must be taken with analyses based on administrative health data to capture all populations eligible for treatment and to understand the contribution of these subpopulations to overall outcomes.

## Background

Accountability and transparency in health care, at individual and institutional levels, has gained increasing importance in recent years [1-3]. This growing focus on quality of practise and safety of services has implications for patients, clinicians, administrators and policy makers. Quality assessments of hospital performance are now conducted routinely throughout the world, the results of which are made available to all stakeholders. Information of this kind has been used for the purposes of benchmarking, so as to improve performance across all institutions [4] and to empower the general public to make informed decisions about where they choose to receive care.

Surgical resection for curable oesophageal and gastro-oesophageal cancer is the mainstay of treatment for all patients who are fit for major surgery. However, despite being the only curative treatment for oesophageal/gastro-oesophageal cancer patients, oesophagectomy is associated with significant operative morbidity and mortality [5-7]. Key performance measures of oesophagectomy include 30-day mortality, hospital length-of-stay and post-surgery complication rate [8-17]. Factors influencing outcomes following oesophagectomy include hospital/surgeon factors (peer group/volume, surgical experience), tumour stage, histology and location, surgery type and patient comorbidity; with better outcomes reported in high volume hospitals, patients with non-metastatic disease (approximately 60% of patients), adenocarcinoma (the incidence of which is increasing), and when transhiatal oesophagectomy is performed [18-25].

In Australia, there has been keen interest in using administrative datasets to examine hospital performance [26-28] with surgeons, administrators and patients having endorsed independent review of surgical care [29]. We previously examined patient outcomes for oesophageal cancer following oesophagectomy in one Australian state using data linkage of administrative datasets [9]. We found oesophagectomy was performed with satisfactory outcomes however resection rates were lower than reported elsewhere, as other studies included cancer of the gastro-oesophageal junction in their analyses since gastro-oesophageal cancer is treated in a similar manner to oesophageal cancer [30,31].

Therefore, our aim was to examine rates of oesophogectomy and post-resection outcomes for patients diagnosed with oesophageal cancer or gastro-oesophageal cancer in the state of New South Wales (NSW) between 2000 and 2007. Specifically, we examined the way in which the inclusion of gastro-oesophageal cancer to the oesophageal cancer cohort impacted on resection rates and post-resection outcomes.

## Methods

### Setting

Australia has a publicly-funded universal health care system and health care data is collected by Commonwealth and state-based jurisdictions. The Australian Government Department of Human Services administer the Medicare Benefits Scheme (MBS; which provides free or subsidised treatment by health professionals and fully subsidised treatment in public hospitals) and the Pharmaceutical Benefits Scheme (PBS; which provides subsidised access to prescription drugs). The individual state and territory health authorities are responsible for the collection of public and private hospital separation data within their jurisdiction. However, cross-jurisdictional data sets have not been linked routinely due to legislative and privacy issues.

Our current study uses routinely collected hospital separation data from New South Wales (NSW). NSW is the largest jurisdiction in Australia with eight area health districts (at the time this research commenced) and consists of more than 200 public and 80 private hospitals, some of which are in proximity to each other (co-located). As such medical specialists practise at both the public and affiliated private hospital.

### Study population

Our study population consisted of patients with a primary invasive oesophageal and gastro-oesophageal cancer (International Classification of Diseases v3 [ICD-O-3] codes C15.0-C15.9 and C16.0).

### Data sources and linkage

We accessed and linked two population-based data sets in this study:

*NSW Central Cancer Registry (1 July 2000-31Dec2007)* - (CCR) receives notifications of cancer in NSW and maintains a record of all cancer cases diagnosed in NSW residents since 1972. The registry is managed according to the International Association of Cancer Registries (IACR) rules [32] and is one of the few Australian Cancer Registries to record degree-of-spread at first diagnosis for all solid malignant tumours [33]. Degree-of-spread is assigned by the NSW CCR into one of four summary stages (localised, regional, distant or unknown) [25]. Histology type was based on Berg groupings of ICD-O-3 classifications and grouped as adenocarcinomas, squamous cell carcinoma (SCC) or other.

*Admitted Patient Data Collection (1 July 2000 – 30 June 2008)* - is a census of all inpatient separations from all public, private and repatriation hospitals, private day procedures centres and public nursing homes in NSW. We identified surgical resections in the separations using the International Classification of Diseases (ICD_10_AM) procedural block codes for oesophagectomy by abdominal and thoracic/cervical/transthoracic mobilisation and endoscopic mucosal resection 0858–0860 (if recorded) [34].

Data linkage was undertaken by a third-party, the NSW Centre for Health Record Linkage (CHeReL) using best practice privacy preserving protocols. Using probabilistic linkage, CHeReL identified all appropriate records from the APDC and CCR. They then assigned unique identifiers (or Project Person Number: PPN) to the APDC and CCR files. We received two individual data files and linked the unit-record data using the unique PPNs.

### Outcomes measures and statistical analyses

We reported oesophagectomy rates for oesophageal cancer, gastro-oesophageal cancer and the combined cohort. As oesophagectomy is most beneficial for, and is associated with, non-metastatic disease and patients with adenocarcinoma, we also examined rates of surgical resection and factors associated with surgical resection and post-surgical outcomes for this sub-group of patients. Multivariate logistic regression was used to determine which factors were associated with (i) receiving an oesophagectomy and (ii) the post-resection outcomes of: longer length of hospital stay (>28 days); 30-day complication and 30-day mortality. Post resection complications arising within 30 days were derived from ICD-10-AM diagnostic codes and included any post-procedural disorders or complications, haemorrhages, pulmonary and cardiac complications.

Cox proportional hazard regression survival analysis was also undertaken to examine one-year survival following oesophagectomy. We followed patients diagnosed with oesophageal cancer or cancer of the gastro-oesophageal junction in the NSW CCR to 31^st^ December, 2007 for death from the cancer.

We did not identify individual hospitals in this study, rather we grouped hospitals by health service (identified as Area 1 – Area 8) and according to fiscal sector (public co-located; private co-located; public non co-located; private non co-located). We used the Charlson Comorbidity Index (CCI) to account for comorbidity before cancer diagnosis [35]. This index is commonly used to adjust for comorbidity and was derived from ICD-10-AM diagnostic codes as recorded in any hospital separation prior to and including the date of cancer diagnosis [36,37]. Other covariates of interest included patient factors of age at diagnosis, gender, Australian or overseas born, tumour location and surgical procedure (where appropriate).

Statistical significance was taken at the p<0.05 level. All statistical analysis was performed using SAS version 11.

### Ethical approval

This research was approved by the NSW Population and Health Service Research Ethics Committee (2010/05/235).

## Results

### Cohort Characteristics (Table 1)

Our cohort comprised 5,024 patients, 3,456 (69%) of whom were diagnosed with oesophageal cancer and 1,568 with gastro-oesophageal cancer.

More than half of oesophageal cancer patients were ≥70 years, 67% were male, 45% were diagnosed with adenocarcinoma, 20% with regional spread and 18% with distant spread-of-disease. The vast majority (90%) of oesophageal cancer patients were defined by the CCI as having no pre-existing comorbidity. Similarly, most gastro-oesophageal patients were ≥70 years and male. Owing to the nature of gastro-oesophageal cancer, 89% of this group were diagnosed with adenocarcinoma, 35% with regional and 25% with distant disease spread. The addition of gastro-oesophageal patients to the cohort increased the proportion of patients in the overall cohort with adenocarcinoma to approximately 60%, and patients with regional or distant spread increased from 38% to 45%.

**Table 1:** Characteristics of patients diagnosed with oesophageal (C15) and gastro-oesophageal cancer (C160)

**Variable**	**C15**		**C160**		**C15 & C160**	
	**N=3,456**		**N=1,568**		**N=5,024**	
	**n**	**%**	**n**	**%**	**n**	**%**
Age at diagnosis group (years)						
<60	657	19.0	402	25.6	1,059	21.1
60–69	840	24.3	399	25.4	1,239	24.7
70–79	1,048	30.3	486	31.0	1,534	30.5
80+	911	26.4	281	17.9	1,192	23.7
Gender						
Female	1,159	33.5	362	23.1	1,521	30.3
Male	2,297	66.5	1,206	76.9	3,503	69.7
Country of Birth						
Australia	2,497	72.2	1,063	67.8	3,560	70.9
Other	959	37.8	505	32.2	1,464	29.1
Histology group						
SCC	1,478	42.8	70	4.5	1,548	30.8
Adenocarcinomas	1,549	44.8	1,398	89.2	2,947	58.7
Other	293	8.5	79	5.0	372	7.4
Degree of Spread						
Localised	1,290	37.3	435	27.7	1,725	34.3
Regional	692	20.0	543	34.6	1,235	24.6
Distant	622	18.0	387	24.7	1,009	20.1
Unknown	721	20.9	203	12.9	924	18.4
Tumour location						
Upper	297	8.6			297	5.9
Middle	470	13.8			470	9.4
Lower	1,528	45.0			1,528	30.9
NOS*	1,113	32.7			1,113	22.2
GOJ*			1,568		1,568	31.2
Charlson comorbidity						
0	3,098	89.6	1,431	91.2	4,529	90.1
1,2	331	9.6	124	7.9	455	9.1
3+	27	0.8	13	0.8	40	0.8

*NOS = not otherwise specified; GOJ = gastro-oesophageal junction.

### Rates of surgical resection and factors associated with receiving surgical resection (Tables 2, 3)

The rate of resection in oesophageal cancer patients was 14.6%, whereas 26.4% of gastro-oesophageal patients underwent surgical resection. The addition of gastro-oesophageal cancer patients to the cohort increased the overall surgery rates to 18.2%.

The majority of oesophageal cancer patients who underwent resection were <70 years (71%), male (76%), born in Australia (68%), had no pre-existing comorbidity (70%) and were diagnosed with adenocarcinoma (62%) and with loco-regional disease at the time of notification (85%). Gastro-oesophageal cancer patients had even higher proportions of males (87%) and adenocarcinoma patients (93%) who underwent resection. After adjustment for covariates, surgical resection for oesophageal cancer patients was associated with younger age (*p*<0.001), adenocarcinoma (*p*<0.001*)*, non-metastatic disease (*p<*0.001), no pre-existing comorbidity (*p*=0.006) and the resection being conducted in a public or private co-located hospital (*p*<0.001) (Table 2). Not surprisingly the addition of gastro-oesophageal patients to the cohort increased patient numbers and resulted in some additional significant predictors of surgical resection (male gender [aOR=1.31 95%CI 1.05-1.64], born in Australia [aOR=1.32, 95%CI 1.09-1.61] and undergoing surgery at specific area health services).

**Table 2:** Patient characteristics, location and surgery type of cohort who received resection

**Variable**	**C15**		**C160**		**C15 & C160**	
	**N=3,456**		**N=1,568**		**N=5,024**	
	**n**	**%**	**n**	**%**	**n**	**%**
Oesophagectomy Yes	503	14.6	412	26.3	915	18.2
PATIENT CHARACTERISTICS						
Age at diagnosis (years)						
<60	164	4.7	137	8.7	301	6.0
60–69	195	5.6	137	8.7	332	6.6
70–79	116	3.4	120	7.7	236	4.7
80+	28	0.8	18	1.1	46	0.9
Gender						
Female	118	3.4	54	3.4	172	3.4
Male	385	11.1	358	22.8	743	14.8
Country of Birth						
Australia	345	10.0	291	18.6	636	12.7
Other	158	4.6	121	7.7	279	5.6
Histology group						
SCC	169	4.9	12	0.8	181	3.6
Adenocarcinomas	312	9.0	385	24.6	697	13.9
Other	11	0.3	11	0.7	22	0.4
Degree of Spread						
Localised	197	5.7	123	7.8	320	6.4
Regional	232	6.7	239	15.2	471	9.4
Distant	38	1.1	35	2.2	73	1.5
Unknown	36	1.0	15	1.0	51	1.0
Tumour location						
Upper	17	0.5			17	0.3
Middle	55	1.6			55	1.1
Lower	331	9.6			331	6.6
NOS*	99	2.9			99	2.0
GOJ*			412	26.3	412	8.3
Charlson comorbidity						
0	353	10.2	284	18.1	637	12.7
1,2	32	0.9	22	1.4	54	1.1
3+	0	0.0	2	0.1	2	0.0
HOSPITAL CHARACTERISTICS						
Surgery type						
Abdothoracic	79	2.3	65	4.1	144	2.9
Abdocervical	64	1.9	45	2.9	109	2.2
Transhiatal	360	10.4	301	19.2	661	13.2
Hospital admission						
Private co-located	97	2.8	75	4.8	172	3.4
Private non co-located	90	2.6	87	5.5	177	3.5
Public co-located	182	5.3	131	8.4	313	6.2
Public non co-located	134	3.9	119	7.6	253	5.0
Hospital area service						
Area 1	99	2.9	90	5.7	189	3.8
Area 2	107	3.1	85	5.4	192	3.8
Area 3	93	2.7	70	4.5	163	3.2
Area 4	126	3.6	90	5.7	216	4.3
Area 5	53	1.5	51	3.3	104	2.1
Area 6	11	0.3	9	0.6	20	0.4
Area 7	14	0.4	17	1.1	31	0.6
Area 8	0	0.0	0	0.0	0	0.0

* NOS = not otherwise specified; GOJ = gastro-oesophageal junction.

**Table 3:** Surgery rates and factors associated with receiving surgery by topography

**Variable**	**C15 Surgical Rate (per 100)**	**aOR**^**#**^**(95%CI)**	**C15&C160 Surgical rate (per 100)**	**aOR (95% CI)**^**#**^
Age at diagnosis (years)				
<60	25.0	8.96 (5.63-14.3)*	28.4	8.12 (5.64-11.7)*
60–69	23.2	9.85 (6.25-15.5)*	26.8	8.48 (5.94-12.1)*
70–79	11.1	3.99 (2.52-6.32)*	15.4	4.20 (2.94-6.00)*
80+	3.1	Referent	3.9	Referent
Gender				
Female	10.2	Referent	11.3	Referent
Male	16.8	1.04 (0.78-1.38)	21.2	1.31 (1.05-1.64)*
Country of Birth				
Australia	13.8	Referent	17.9	Referent
Other	16.5	0.90 (0.70-1.16)	19.1	0.76 (0.62-0.92)*
Histology group				
SCC	11.4	0.40 (0.31-0.52)*	11.7	0.41 (0.32-0.51)*
Adenocarcinomas	20.1	Referent	23.6	Referent
Other	3.8	0.28 (0.17-0.48)*	5.9	0.36 (0.24-0.53)*
Degree of Spread				
Localised	15.3	6.06 (4.06-9.06)*	18.6	5.50 (4.08-7.41)*
Regional	33.5	12.34 (8.22-18.52)*	38.1	10.29 (7.67-13.79)*
Distant	6.1	Referent	6.4	Referent
Unknown	5.0	2.38 (1.42-3.98)*	5.5	1.71 (1.14-2.56)*
Tumour location				
Upper	5.8	0.22 (0.12-0.39)	5.8	0.22 (0.13-0.40)
Mid	10.9	0.51 (0.34-0.76)	10.9	0.52 (0.35-0.77)
Distal	21.5	Referent	21.5 26.4	Referent 1.10 (0.88-1.36)
Charlson comorbidity				
0	11.4	Referent	14.1	Referent
1,2	9.7	0.52 (0.32-0.83)*	11.9	0.56 (0.39-0.80)*
3+	0	-	5.0	0.25 (0.06-1.14)
Hospital admission				
Private co-located	43.1	Referent	46.5	Referent
Private non co-located	4.4	0.06 (0.04-0.10)*	6.2	0.07 (0.05-0.10)*
Public co-located	34.0	0.78 (0.53-1.16)	37.8	0.80 (0.59-1.08)
Public non co-located	20.6	0.26 (0.16-0.42)*	26.5	0.26 (0.18-0.38)*
Hospital area service				
Area 1	18.1	2.30 (1.38-3.84)*	23.6	2.99 (2.03-4.39)*
Area 2	16.2	0.73 (0.48-1.09)	19.4	0.80 (0.59-1.09)
Area 3	25.8	Referent	28.6	Referent
Area 4	19.6	1.29 (0.86-1.93)	23.8	1.58 (1.16-2.17)*
Area 5	12.2	1.71 (0.99-2.97)	16.5	1.92 (1.27-2.90)*
Area 6	3.5	0.82 (0.38-1.77)	4.6	0.88 (0.49-1.56)
Area 7	5.1	0.71 (0.34-1.47)	8.2	0.98 (0.59-1.65)
Area 8	0.0	-	0.0	-

^#^*Adjusted for age-group, gender, histology, country of birth, histology, degree-of-spread, tumour location, hospital admitted, area of service, Charlson comorbidity index.*

* Significant at P<0.05 (two-tailed) level.

### Sub-analyses of patients with non-metastatic adenocarcinoma

When restricted to the 2,155 non-metastatic adenocarcinoma patients, the addition of gastro-oesophageal cancer patients increased the overall surgery rate to 29.6% (from 26.1% for oesophageal cancer alone). Patient and hospital factors associated for surgery in this sub-cohort were the same as the entire cohort. The addition of the gastro-oesophageal patients again added the significant predictors of surgical resection (male gender [aOR=2.03 95%CI 1.44-2.85], being born in Australia [aOR=1.41, 95%CI 1.35-3.44] and undergoing surgery at specific area health services).

### Outcomes following surgery (Table 4)

**Table 4:** Patient and hospital factors associated with outcomes following surgical resection

	**C15**		**C15 & C16.0**	
**Outcome**	**N (Rate per 100)**	**aOR (95%CI)**^**#**^	**N (Rate per 100)**	**aOR (95%CI)**^**#**^
(**i) Length-of-stay >28 days**	**105 (20.9)**		**186 (19.7)**	
<60 years	23 (14.0)	0.20 (0.08-0.53)*	43 (14.3)	0.24 (0.12-0.51)*
60–64 years	43 (22.1)	0.41 (0.17-1.00)	67 (20.2)	0.43 (0.22-0.87)*
70–74 years	28 (24.1)	0.44 (0.17-1.13)	60 (25.4)	0.57 (0.28-1.17)
80+ years	11 (39.3)	Referent	16 (34.8)	Referent
Australian born	75 (21.7)	Referent	133 (20.9)	Referent
Overseas born	30 (19.0)	0.62 (0.38-1.05)	53 (19.0)	0.67 (0.46-0.99)*
Private co-located	14 (14.4)	Referent	22 (12.8)	Referent
Private non co-located	11 (12.2)	1.06 (0.43-2.63)	23 (13.0)	1.92 (0.90-4.13)
Public co-located	43 (23.6)	1.61 (0.74-3.49)	71 (22.7)	2.10 (1.17-3.78)*
Public non co-located	37 (27.6)	3.16 (0.98-10.8)	70 (27.7)	7.89 (3.16-19.5)*
Area 1	29 (29.3)	0.91 (0.26-3.18)	52 (27.5)	0.44 (0.17-1.10)
Area 2	22(20.6)	0.91 (0.43-1.96)	43 (22.4)	1.24 (0.70-2.19)
Area 3	22 (23.7)	Referent	37 (22.7)	Referent
Area 4	21 (16.7)	0.74 (0.33-1.66)	33 (15.3)	0.64 (0.34-1.20)
Area 5	8 (15.1)	0.30 (0.07-1.26)	14 (13.5)	0.15 (0.05-0.43)*
Area 6	2 (18.2)	0.82 (0.12-5.59)	3 (15.0)	0.30 (0.07-1.39)
Area 7	1 (7.1)	0.18 (0.02-1.92)	4 (12.9)	0.22 (0.06-0.86)*
(**ii) 30 day complication**	**108 (21.5)**		**168 (17.0)**	
<60 years	35 (21.3)	0.40 (0.17-0.98)*	53 (17.6)	0.53 (0.26-1.10)
60–64 years	36 (18.5)	0.33 (0.14-0.80)*	59 (17.8)	0.55 (0.27-1.13)
70–74 years	25 (21.6)	0.39 (0.16-0.99)*	52 (22.0)	0.70(0.34-1.46)
80+ years	12 (42.9)	Referent	14 (30.4)	Referent
Australian born	71 (20.6)	Referent	129 (20.3)	Referent
Overseas born	37 (23.4)	1.28 (0.79-2.08)	49 (17.6)	0.87 (0.92-1.06)
Private co-located	16 (16.5)	Referent	28 (16.3)	Referent
Private non co-located	22 (24.4)	1.13 (0.43-2.98)	32 (18.1)	1.07 (0.51-2.23)
Public co-located	40 (22.0)	1.82 (0.87-3.78)	61 (19.5)	1.37 (0.79-2.40)
Public non co-located	30 (22.4)	0.74 (0.23-2.37)	57 (22.5)	1.47 (0.62-3.49)
Area 1	25 (25.3)	3.88 (1.12-13.5)*	40 (21.2)	1.36 (0.54-3.47)
Area 2	21 (19.6)	1.91 (0.85-4.28)	37 (19.3)	1.40 (0.76-2.57)
Area 3	15 (16.1)	Referent	28 (17.2)	Referent
Area 4	29 (23.0)	2.24 (0.99-5.09)	40 (18.5)	1.29 (0.68-2.42)
Area 5	13 (24.5)	4.01 (1.04-15.4)*	23 (22.1)	1.34 (0.49-3.65)
Area 6	9 (18.2)	2.86 (0.43-19.1)	2 (10.0)	0.74 (0.14-3.97)
Area 7	11 (21.4)	3.87 (0.68-22.1)	8 (25.8)	2.14 (0.66-6.91)
	Mortality N (rate per 100)	aHR (95%CI)	Mortality N (rate per 100)	aHR (95%CI)
**(iv) One year survival**	**123 (24.5)**		**218 (23.1)**	
<60 years	36 (22.0)	0.20 (0.10-0.41)*	64 (21.3)	0.41 (0.23-0.74)*
60–64 years	38 (30.9)	0.19 (0.09-0.38)*	65 (19.6)	0.36 (0.20-0.65)*
70–74 years	32 (27.6)	0.22 (0.10-0.47)*	68 (28.8)	0.44 (0.24-0.81)*
80+ years	17 (60.7)	Referent	21 (45.6)	Referent
Australian born	90 (26.1)	Referent	155 (24.4)	Referent
Overseas born	33 (20.9)	0.64 (0.39-1.06)	63 (22.6)	0.74 (0.51-1.08)
Private co-located	10 (10.3)	Referent	20 (11.6)	Referent
Private non co-located	22 (24.4)	1.58 (0.57-4.42)	40 (22.6)	2.20 (1.03-4.70)*
Public co-located	48 (26.4)	1.25 (0.55-2.83)	80 (25.6)	1.80 (0.97-3.32)
Public non co-located	43 (32.1)	1.34 (0.45-4.02)	78 (30.8)	1.66 (0.71-3.89)
Area 1	34 (34.3)	1.06 (0.36-3.16)	61 (32.3)	1.28 (0.54-3.03)
Area 2	23 (21.5)	0.93 (0.42-2.04)	41 (21.4)	1.08 (0.60-1.96)
Area 3	25 (26.9)	Referent	38 (23.3)	Referent
Area 4	19 (15.1)	0.73 (0.32-1.68)	38 (17.6)	0.93 (0.49-1.76)
Area 5	15 (28.3)	0.85 (0.26-2.82)	30 (28.9)	1.67 (0.67-4.11)
Area 6	2 (18.2)	1.52 (0.27-8.52)	5 (25.0)	1.92 (0.59-6.22)
Area 7	5 (35.7)	1.45 (0.36-5.87)	5 (16.1)	0.76 (0.22-2.66)

^#^* Adjusted for age-group, gender, histology, country of birth, degree-of-spread, tumour location, hospital admitted, area of service, Charlson comorbidity index, surgery type.*

* Significant at the P<0.05 (two-tailed) level.

Rates of hospital length-of-stay >28 days (20.9% and 19.7%), 30-day mortality (3.8% and 2.7%) and one-year cancer survival (75.5% and 76.9%) were similar for oesophageal cancer and the combined cohort respectively, but differed by 4.5% for 30-day complication rates (21.5% and 17.0%). Mean one year survival time for the combined cohort was 328 days (SD 48 days).

When investigating the factors associated with longer length of hospital stay and one-year survival, the inclusion of cancer of the gastro-oesophageal junction did not alter the trends established with that of oesophageal cancer alone. However, due to increased precision, some of the associations that were not statistically significant in oesophageal cancer alone became significant in the overall cohort. For longer length of stay these factors were: being Australian born (aOR=1.49, 95%CI 1.01-2.17) and having surgery in a public co-located hospital (aOR=2.10, 95%CI 1.17-3.78). Other factors associated with length of stay >28 days included older age (*p*<0.001), having an admission in a public hospital (p<0.001) and not being admitted in certain area health services. Poorer survival in the overall cohort was associated with older age (*p*<0.001) and private non-co-located hospitals compared with private co-located hospitals (aHR=2.20, 95%CI 1.03-4.70).

We did not observe any significant associations for 30 day post-resection complication in the overall cohort. However, in the oesophageal cohort we found older age and some area health services were associated with increased complication rates. The loss of statistical significance in the overall cohort may be attributed to the lower complication rate with gastro-oesophageal cancer.

The small number of patients dying within 30-days of surgery did not permit us to perform adjusted analysis for this outcome measure.

### Outcomes following surgery in patients with non-metastatic adenocarcinoma

Rates of hospital length-of-stay >28 days (17.3% and 19.6%), 30-day mortality (2.4% and 2.8%), 30-day complication rates (19.0% and 19.7%) and one-year cancer survival (80.6% and 79.7%) were similar for patients with non-metastatic adenocarcinoma oesophageal cancer and the combined cohort respectively.

Factors associated with poorer post-resection outcomes in the sub-cohort were similar to those reported in all patients. However, in this group of patients with non-metastatic adenocarcinoma, we did not observe any significant associations for 30-day post-resection complication in the combined cohort or in oesophageal cancer alone.

## Discussion

There is an increasing use of health administrative data for research and in health policy and planning. We examined rates of oesophagectomy on oesophageal cancer patients and whether the inclusion of patients with gastro-oesophageal cancer affected oesophagectomy rates and post-resection outcomes in NSW. Unsurprisingly, the addition of gastro-oesophageal cancer patients to our cohort increased the oesophagectomy rates by 3.5%. Clearly if hospital funding is based partly on patient admissions and the volume of procedures undertaken, care must be taken to ensure that all potential ‘qualifying’ patients are included in any performance assessment. Our reported rate of oesophagectomy in patients is comparable to previously reported European and UK studies [30,31] However, the UK rate has been decreasing [38] and may be due to the highly specialised pre- and peri-operative assessment and management required for conducting an oesophagectomy [39,40], and the support for centralization of oesophagectomies being performed in dedicated multidisciplinary centres [41,42].

Due to increased precision, the inclusion of gastro-oesophageal cancer resulted in an increase in the number of predictor variables associated with rates of resection and post-resection outcomes that were not seen with oesophageal cancer alone (with the exception of 30-day complication rates). For our overall cohort, factors associated with receiving an oesophagectomy included patient characteristics (age, gender, birthplace) cancer characteristics (histology, disease spread) and hospital related factors (location and funding sector); similar to previously published work [31]. 30-day mortality in this study was low, in-line with previous studies and international benchmarks [9,38,43]. We found that patients undergoing surgical resection in public hospitals were more likely to have a length of stay >28 days. This may reflect the epidemiology of oesophageal and gastro-oesophageal cancer and that more complex cases are treated in public hospitals (teaching hospitals linked to academic centres in Australia). Thus, the addition of gastro-oesophageal cancer did impact on factors associated with receiving an oesophagectomy and on some post-surgery outcomes.

One-year cancer survival was worse in those who underwent surgery in private hospitals that were not co-located with public hospitals, potentially indicating the benefits of surgery being conducted in higher volume or teaching hospitals [7,9,18,21,30,41,44,45].

Our current study demonstrated a reduced 30-day complication rate in gastro-oesophageal cancer compared with oesophageal cancer, hence statistical associations tended towards the null in the combined cohort. This may have been due to the slightly higher rate of transhiatal oesophagectomies undertaken for adenocarcinoma, compared with that in oesophageal cancer and hence lower peri-operative morbidity [46], however the only factors which were significantly associated with higher complication rate in patients with oesophageal cancer were older age and resection being conducted in some area health services.

Although we attempted to account for comorbidity with the use of the CCI, the proportion of patients with comorbidity preceding cancer diagnosis was low and as aforementioned, due to how CCI is calculated, comorbidity was most likely under-ascertained. The under-reporting of comorbidities in administrative data may be due to incomplete data transposition from medical records in individual hospitals to administrative databases [47-49]. Further, non-surgical treatments such as chemotherapy and radiotherapy are funded by the PBS and MBS respectively and this data is not linked routinely to hospital morbidity data thus it was not available for this population linkage study. Further, the only measure of disease severity available was degree-of-spread at diagnosis; there were no indicators of performance status available.

## Conclusion

Oesophagectomy in NSW is performed with good outcomes. Our own experience highlights the need for identifying the appropriate patient populations under study and their unique contributions to the overall cohort, given the change in surgical resection rate and longer length-of-stay post-resection with the inclusion of gastro-oesophageal cancer. The measurement of hospital performance is a powerful tool to stimulate quality improvement, transparency and accountability and health administrative data sets have become pivotal to this activity.

## Competing interests

The authors declare that they have no competing interests.

## Authors' contributions

ES helped to design the study, conducted the statistical analysis and drafted the manuscript. SAP and RW designed the study and helped to draft the manuscript. All authors read and approved the final manuscript.

## Pre-publication history

The pre-publication history for this paper can be accessed here:

http://www.biomedcentral.com/1472-6963/12/384/prepub
